# CIRSE Standards of Practice on Management of Endoleaks Following Endovascular Aneurysm Repair

**DOI:** 10.1007/s00270-023-03629-1

**Published:** 2024-01-12

**Authors:** Joo-Young Chun, Michiel de Haan, Geert Maleux, Asaad Osman, Alessandro Cannavale, Robert Morgan

**Affiliations:** 1 St George’s University Hospitals NHS Foundation Trust, London, UK; 2 St George’s University of London, London, UK; 3 Maastricht University Medical Center, Maastricht, The Netherlands; 4 University Hospitals Leuven, Louvain, Belgium; 5 Policlinico Umberto, Rome, Italy

**Keywords:** Endovascular aneurysm repair, Endoleak types, Imaging, Surveillance, Indication for treatment, Endovascular, Embolisation

## Abstract

**Background:**

Endoleaks represent the most common complication after EVAR. Some types are associated with ongoing risk of aneurysm rupture and necessitate long-term surveillance and secondary interventions.

**Purpose:**

This document, as with all CIRSE Standards of Practice documents, will recommend a reasonable approach to best practices of managing endoleaks. This will include imaging diagnosis, surveillance, indications for intervention, endovascular treatments and their outcomes. Our purpose is to provide recommendations based on up-to-date evidence, updating the guidelines previously published on this topic in 2013.

**Methods:**

The writing group was established by the CIRSE Standards of Practice Committee and consisted of clinicians with internationally recognised expertise in endoleak management. The writing group reviewed the existing literature performing a pragmatic evidence search using PubMed to select publications in English and relating to human subjects up to 2023. The final recommendations were formulated through consensus.

**Results:**

Endoleaks may compromise durability of the aortic repair, and long-term imaging surveillance is necessary for early detection and correct classification to guide potential re-intervention. The majority of endoleaks that require treatment can be managed using endovascular techniques. This Standards of Practice document provides up-to-date recommendations for the safe management of endoleaks.

## Introduction

The CIRSE Standards of Practice Committee established a writing group, which was tasked with producing up-to-date recommendations for the management of endoleaks following aortic endovascular aneurysm repair (EVAR). CIRSE Standards of Practice documents are not clinical practice guidelines or systematic reviews of the literature; they are not intended to impose a standard of clinical patient care but to recommend a reasonable approach to and best practices for performing endoleak repair. Institutions should regularly review their internal procedures for development and improvement, taking into account international guidance, local resources and regular internal morbidity and mortality reviews.

## Methods

The writing group, which was established by the CIRSE Standards of Practice Committee, consisted of six clinicians with internationally recognised expertise in the management of endoleaks. The writing group reviewed existing literature on endoleak repair, performing an extensive evidence review to search for relevant publications in the English language from 1998 to date. Evidence reviewed included guidelines, trials, systematic reviews, and registries, taking into account data on novel techniques, devices, and long-term outcomes that have emerged over the last decade. The writing group formulated the recommendations during three teleconferences and one in-person meeting at the CIRSE Annual Congress 2022.

## Background

An endoleak (EL) is defined as persistent blood flow in the aneurysm sac outside the stent graft after aortic endovascular aneurysm repair (EVAR). They represent the most common complication after EVAR with an incidence of 10–50% [1]. Some types are associated with ongoing risk of aneurysm rupture, necessitating long-term surveillance and secondary interventions [2–4].

ELs may be classified into primary (present at the time of repair or within 30 days of EVAR) or secondary (occurring after previous negative imaging or beyond 30 days). There are five types of EL based on their anatomical site and aetiology (Table 1). Management depends on EL type and the associated risk of sac rupture [1, 4–11].

**Table 1:** Summary of endoleak type and reported incidence [1, 9, 11]

Endoleak type	Location of Leak	Incidence (%)
Type I	Attachment sites	2–10
A	Proximal end	
B	Distal end	
C	Iliac occluder	
Type II	Retrograde flow through patent aortic side branches	8–29
A	Single vessel	
B	Multiple vessels	
Type III	Mechanical failure	1–5
A	Modular disconnection	
B	Fabric tear	
C	Junctional separation (fenestration, branch, visceral stent)	
Type IV	Graft porosity	< 1
Type V	Aneurysm sac enlargement without visualised endoleak	2–3

The EUROSTAR registry demonstrated that type I and III ELs increase the risk of aneurysm rupture (Table 2) and subsequent studies have identified type I and III ELs to most commonly associated with late rupture, responsible for over 60% of sac ruptures [12, 13] (Table 3). Furthermore, a review of EVAR 1 and 2 trial data showed type II EL with sac expansion to be a risk factor for late aneurysm rupture [14].

**Table 2:** Endoleak and aneurysm rupture risk (Adapted from EUROSTAR experience [4])

Endoleak type	Number of patients	Number of late ruptures (%)	Cumulative rupture risk at 2y (%)
I or III	297	10 (3.4)	4.0
II	191	1 (0.5)	1.8
No EL	1,975	5 (0.25)	0.7

**Table 3:** Reasons for late aneurysm rupture post-EVAR [12, 13]

Cause of rupture	% of ruptures	
	N = 235 [12]	N = 190 [13]
I	37.4	52.1
II	9.8	7.4
III	11.1	13.7
IV	0	0.5
V	3.8	1.6

Therefore, early detection and correct classification of ELs is vital to plan optimal management. Once detected, ELs that require treatment are managed predominantly with endovascular techniques.

## Imaging of Endoleaks

Imaging surveillance is necessary in all patients who undergo EVAR to identify complications including EL, aneurysm sac growth and stent graft migration. The rationale for regular imaging surveillance in the first 5 years after EVAR reflects the significant incidence of complications in this postoperative period [10]. Many centres continue lifelong surveillance, while recognising that this increases the cost of aortic repair by 50% and results in a higher radiation burden for patients [1].

A typical surveillance protocol includes a computed tomography (CT) scan at 1 month after EVAR and at 12 months. Further surveillance with duplex ultrasound (US) rather than CT is considered safe if the CT imaging at 1 year shows no EL and stable sac size, or a type II EL with stable sac size. However, an additional CT at 6 months should be considered if the 1-month scan shows a type I or III EL or (unexplained) aneurysm sac expansion [10, 15–17]. Also, detection of a new EL or of aneurysm sac growth of over 5-10 mm on an annual duplex US should prompt further evaluation with CT [1, 10, 16, 18].

Although the mainstay of imaging follow-up relies on CT and US, recent studies and guidelines have highlighted the role of contrast-enhanced US (CEUS) and MR angiography (MRA) in EL surveillance [10, 15, 19, 20]. The combined approach of US, CT and MR can detect up to 91% of ELs [15]. Factors such as patient habitus, EL type, and the local costs and availability of imaging modalities all play a role in deciding optimal imaging follow-up protocols.

**Fig. 1:**
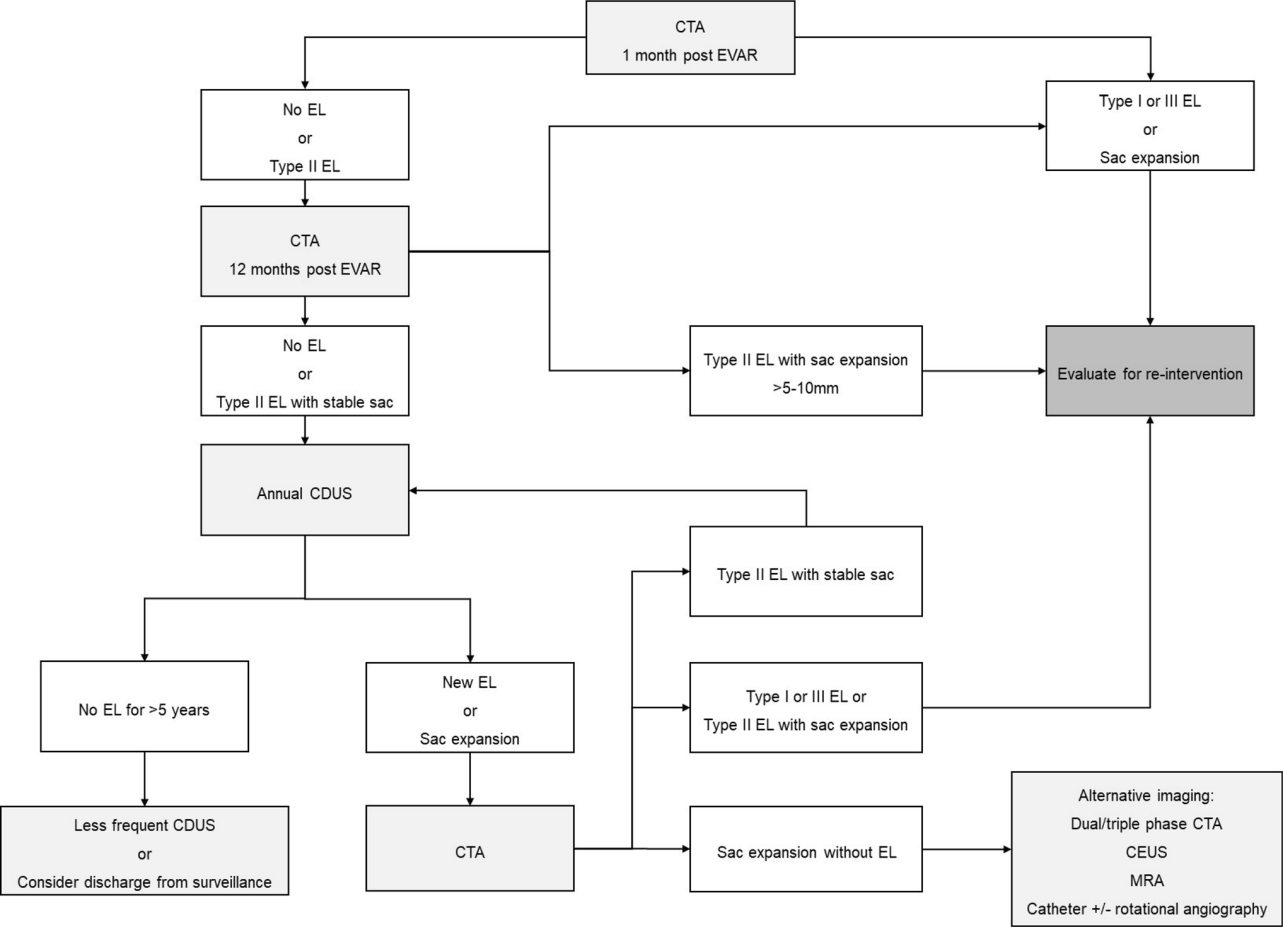
Suggested surveillance protocol for endoleak

A suggested surveillance protocol is outlined in Fig. 1 based on current recommendations.

### Computed Tomography Angiography (CTA)

CTA remains the imaging modality of choice in EVAR surveillance and the mainstay of many surveillance protocols. The main drawback of CT is the cumulative radiation dose, which may be significant over lifelong surveillance. A single-phase arterial CT study may be sufficient to depict ELs for standard EVAR follow-up, with delayed phase + / − pre-contrast imaging reserved for problem-solving [1, 11]. It is also important to monitor renal function prior to administration of iodinated contrast to minimise the risk of contrast-induced nephropathy.

Alternatively, advanced CT protocols may be adopted that can generate more than one phase of imaging from a single acquisition, thereby optimising EL visualisation while minimising radiation exposure. Dual-energy CT allows post-processing of CTA datasets to create virtual non-contrast images while reducing beam-hardening artefact from stent graft struts and embolic agents [21]. The split-bolus technique involves intravenous contrast injection in two sequential boluses separated by a time delay, which captures both arterial and venous phase images in a single acquisition. The two protocols may be combined and the resulting dual-energy CTA with split-bolus technique would generate triple phase images from a single acquisition [22].

### Colour Duplex Ultrasound (CDUS) and Contrast-Enhanced US (CEUS)

Avoidance of ionising radiation and potentially nephrotoxic contrast agents are the main advantages of CDUS compared with CTA. CDUS also provides dynamic information of ELs, such as flow velocity and direction within the aneurysm sac. Both CDUS and CEUS are accurate in detecting type I and type III ELs as well as sac enlargement [18, 23–25], and CEUS has been shown to increase the sensitivity and specificity of CDUS [15, 26, 27]. Several recent studies have suggested CEUS is as sensitive as CTA in detecting ELs and has higher sensitivity for detection of delayed type II ELs [24, 28]. It remains common practice in many institutions to use CDUS in combination with plain films as the mainstay of imaging surveillance.

CDUS and CEUS may be considered as an alternative to CT in stable aneurysms but only in suitable patients with normal body habitus and minimal bowel gas [1, 10, 29]. Other limitations of US include inter-operator variability and an inability to assess the stent graft for migration, seal zones and integrity.

CEUS is often used in conjunction with CTA to further characterise and assess the flow dynamics of a confirmed EL with US contrast. It is also useful in cases of sac expansion with a negative CTA where the contrast may demonstrate sac reperfusion from a slow type II EL [1].

### Magnetic Resonance Imaging (MRI)

MRI is used less commonly for EL detection after EVAR. Although the sensitivity of gadolinium-enhanced MRI may be superior to CTA for detection of type II and occult ELs [20, 30, 31], image quality and interpretation are hampered by susceptibility artefact from metallic stent grafts and other sources of metal such as embolisation coils and surgical clips [11, 32, 33]. Nitinol stent grafts are generally MR-compatible as they contain titanium, but the nickel component may still cause some imaging artefact. Many commonly used stent grafts contain MR-incompatible metals including stainless steel, cobalt chromium and elgiloy. MRI should be avoided in these cases as the degree of artefact would render the images non-diagnostic. Further limitations to widespread application of MRI in routine follow-up protocols are prolonged examination time and restricted availability due to high costs [11].

Given the greater sensitivity of MRI for type II and occult ELs and considering the limits of MRI, there is a potential role for MRI in cases of sac expansion without an obvious EL on CTA [31, 33]. Blood pool contrast agents, also known as intravascular contrast agents, remain in the bloodstream for a longer period, increase the signal-to-noise ratio, and ultimately improve image resolution. MRI with these agents may increase the positive yield of slow-flow or occult ELs [33, 34].

### Digital Subtraction Angiography (DSA)

Conventional catheter angiography with or without cone-beam CT may be used as a problem-solving tool to determine the type and source of an already detected EL on CTA [1, 11]. For example, type I ELs occurring at the seal zones may be confused with type II EL. Aortography performed with the diagnostic catheter placed above and within the stent graft allows exclusion of a type I EL. Additionally, selective angiography of the superior mesenteric artery (SMA) and iliolumbar arteries allows delineation of the collateral pathways to inferior mesenteric artery (IMA) or lumbar arteries, respectively, as a prelude to embolisation of the type II EL at the same session if suitable. Rotational angiography (catheter angiography with cone-beam CT) may also be useful in cases of sac size increase without a visible EL on non-invasive imaging, although these cases are often challenging to diagnose [11].

### Plain Radiography

Plain radiographs in anteroposterior and lateral projections were previously used routinely to assess for migration, stent fractures and modular separations that may result in type I or III ELs. However, as they do not image ELs directly and are limited in the detection of other complications, they are not suitable as the sole imaging modality for surveillance. Many centres no longer use plain radiographs in their follow-up imaging protocols [1, 9].

## Type I Endoleak 

### Definition

Type I EL is defined as a leak at the attachment site of a stent graft, and a manifestation of sealing failure. Type I ELs are further classified into type IA, IB, and IC depending on the occurrence at the proximal or distal ends of the endograft, or iliac occluder, respectively. Type I EL has been reported to occur in as many as 10% of EVAR cases [2, 35–40] and appear to increase with time from less than 5% incidence on surveillance imaging at 30 days to 6.8% incidence at 12 months [38].

Type IA EL is associated with adverse proximal neck anatomy pre-EVAR, including short (> 15 mm), angulated (> 60°), large diameter (> 32 mm), conical or tapered necks or those with calcification or thrombus [1, 10, 41–44]. Patients with hostile neck anatomy often require adjunctive procedures to achieve an adequate proximal seal and are associated with a four-fold increased risk of developing type IA EL [45]. The use of EVAR in challenging necks outside the device instructions for use (IFU) is associated with a higher incidence of type IA EL, which may contribute to delayed rupture and poor outcomes [46]. In view of this, such practice is discouraged.

Type IB EL is associated with large diameter common iliac arteries > 14 mm, short iliac sealing zones and iliac artery tortuosity [47, 48]. Around 50% occur within 6 months after EVAR and iliac artery expansion at the landing zones in the early postoperative months may result in a loss of seal.

### Indication for Treatment

Type I EL is associated with elevated sac pressure and an ongoing risk of aneurysm expansion and rupture [2, 3, 12, 14, 49–51]. It is the leading cause of late aneurysm rupture in up to 52% of cases [13] and should be treated promptly upon detection [1, 9–11].

### Management

#### Primary Type IA

Intraprocedural type IA EL can be treated with repeated balloon moulding or placement of a giant bare metallic stent at the proximal neck. If additional landing zone length exists, a short aortic cuff may be used to extend the seal zone.

EndoAnchors (Aptus Heli-FX EndoAnchor System, Medtronic, USA) staple the stent graft to the aortic wall by means of a metallic tack. They may be used as an adjunct to prevent type IA EL and stent graft migration in challenging aortic necks or to treat a visible EL [52]. The only comparable controlled data did not show a significant difference in the rate of type 1A EL between stapled and non-stapled cases [53].

#### Secondary Type IA

Delayed type IA EL may occur due to changes in the configuration of the aorta that result in aortic neck dilatation or stent graft migration. Multiple options for re-intervention are available including balloon moulding of the proximal seal zone and placement of bare stents to increase the radial strength at the proximal attachment site.

In selected patients with a suitable landing zone, proximal extension of the stent graft may be considered by means of a simple cuff, either alone or in combination with parallel chimney grafts. Patients with more challenging anatomy may require proximal extension using custom-made fenestrated or branched devices.

**Fig. 2:**
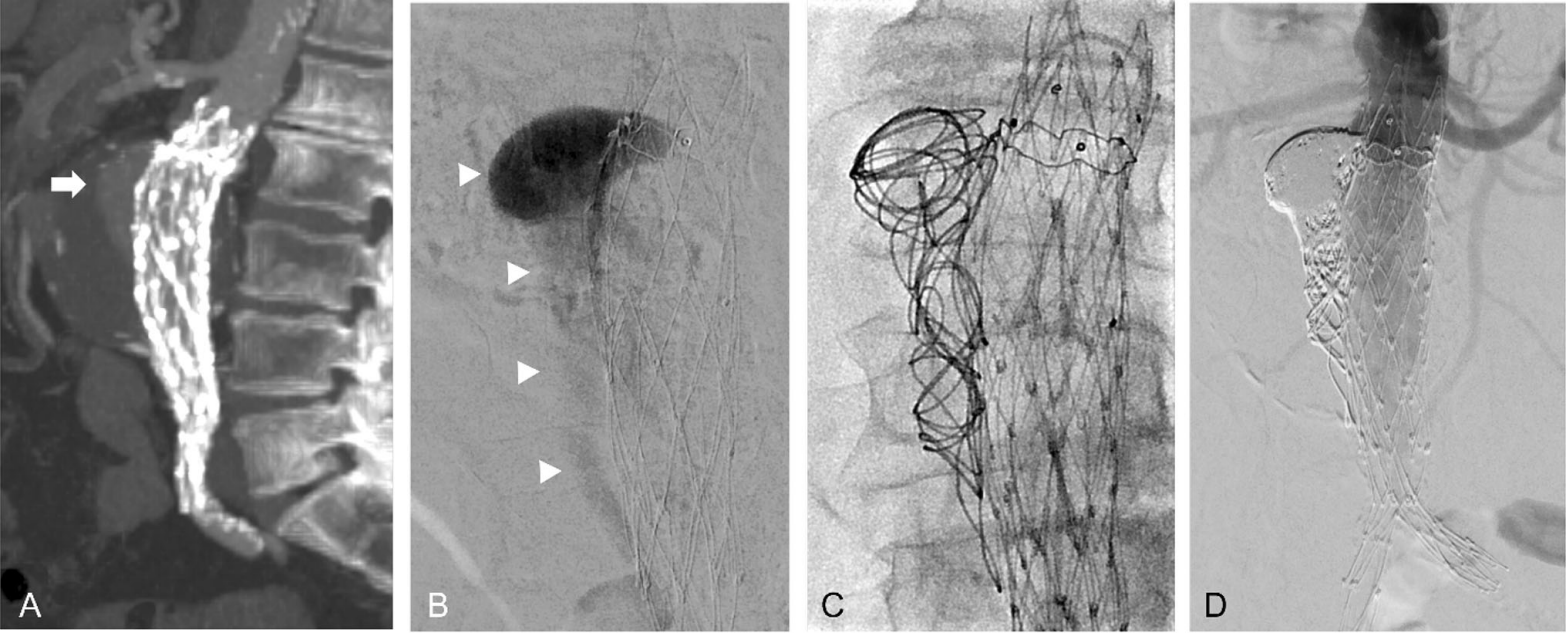
Type IA EL embolisation. (**A**) Sagittal CT image shows proximal type I EL post-EVAR (arrow). Unsuitable for treatment with an aortic cuff due to heavily diseased iliac arteries. (**B**) Entry channel between aortic wall and stent graft was engaged with a reverse-curve catheter. The subsequent angiogram outlines a large EL cavity (arrows). (**C**) Embolisation of EL cavity with coils via a microcatheter. (**D**) Completion angiogram shows successful embolisation

In patients where these techniques have failed, where there is insufficient neck length for stent graft extension, or where the patient is unfit for more complex therapies, transcatheter embolisation is an alternative EL treatment option [1, 10, 11] (Fig. 2). The entry channel between the aortic wall and stent graft is engaged with a guide catheter from a femoral, brachial or radial approach. A microcatheter is introduced coaxially into the EL cavity and an angiogram is performed to assess the size and extent of the EL cavity, the size of the entry channel (neck), and any exiting vessels. Embolisation can be performed with endovascular coils, liquid embolics such as n-butyl cyanoacrylate (NBCA), or ethylene vinyl alcohol copolymer (EVOH) (Onyx, Medtronic, USA), thrombin or a combination of embolic agents.

When endovascular techniques have failed to control a type IA EL, conversion to an open surgical approach may be the only option [1, 9].

#### Type IA in Chimney-EVAR (Ch-EVAR)

Primary type IA EL is common after Ch-EVAR occurring in up to 30% of patients, but the majority resolve spontaneously by 12 months and they are not associated with aneurysm sac growth [54]. About 3% require re-intervention for persistent EL and these pose a challenge as standard treatment options to optimise the proximal seal may not be feasible. If simple endovascular techniques such as simultaneous ballooning of the stent graft and chimneys do not control the EL, embolisation techniques should be considered [11].

#### Type IB

**Fig. 3:**
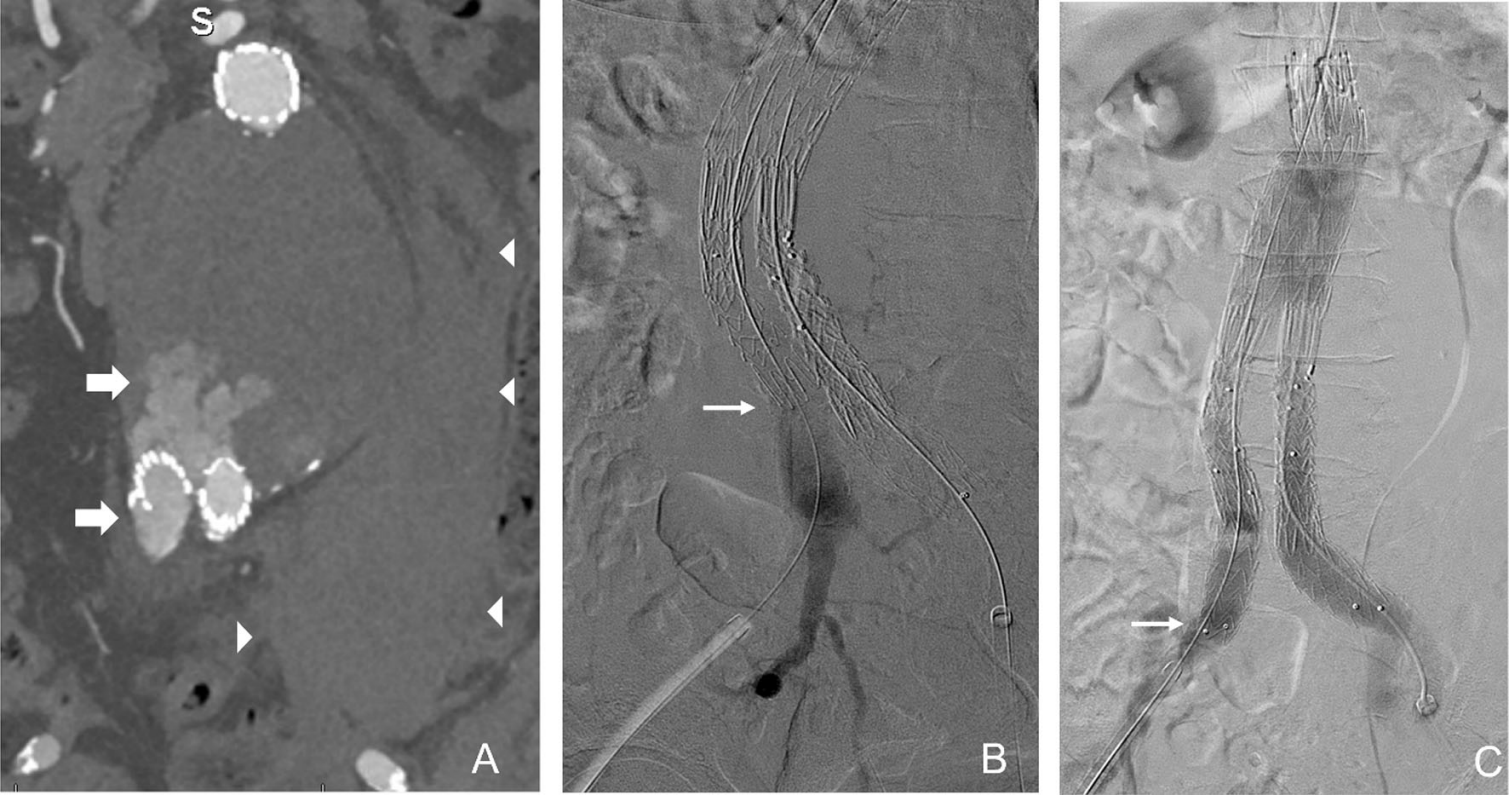
Type IB EL. (**A**) Coronal CT image shows a large type IB EL from a short right iliac limb (arrow). Patient presented with aneurysm rupture (arrowheads). (**B**, **C**) Angiograms before and after successful limb extension. Arrows point to distal extent of right iliac limb

Type IB EL is treated with distal extension of the iliac limb to achieve an adequate distal seal (Fig. 3). If there is insufficient length before the origin of the internal iliac artery, it may be necessary to extend the stent graft into the external iliac artery. The internal iliac artery (IIA) can be over stented, embolised with coils or plugs, or preserved with an iliac branch device or parallel grafts [10, 11, 29].

#### Type IC

EVAR with an aorto-uni-iliac (AUI) device is much less commonly performed than previously and type IC EL is rarely reported. Treatment involves placing additional plugs or coils to achieve complete occlusion of the contralateral common iliac artery.

## Type II Endoleak

### Definition

Type II ELs are caused by retrograde blood flow into the aneurysm sac from aortic or iliac branch arteries, such as lumbar, inferior mesenteric (IMA), accessory renal, median sacral and internal iliac arteries [1, 55]. They are further classified into type IIA EL when only one branch is involved, and type IIB EL when there are two or more branches, usually with a dominant inflow artery and one or more outflow arteries [11]. Type II ELs are the most common EL following EVAR and a major cause for re-intervention. They occur in up to 20–30% of cases [2, 38, 39, 56–58]. Approximately 50% resolve spontaneously in the first 6 months, 5–10% persist beyond 6 months and new type II ELs develop in 5–10% [11, 56, 59, 60] of patients. Factors that increase the risk of developing type II EL include a patent IMA > 3 mm in diameter, a lumbar artery > 2 mm, an aorto-iliac aneurysm and/or a significant mural thrombus burden [57, 61–66].

### Indication for Treatment

There is debate regarding the clinical significance of type II EL and the threshold for intervention. Some authors suggest a more conservative approach as the ELs are inherently low-flow and often transient. The risk of aneurysm rupture in the presence of an isolated type II EL has been shown to be less than 1% [56] and type II EL has not been associated with reduced patient survival [2]. Similarly, several studies have found no differences in aneurysm-related mortality or sac expansion between patients who were treated conservatively for type II EL and those who underwent re-intervention [67, 68].

However, other authors have presented contradictory findings and have demonstrated that some type II ELs may not be benign and are associated with adverse outcomes. For example, type II EL has been shown to be an independent risk factor for sac expansion [69–71] and persistent type II EL to be associated with a higher incidence of re-intervention, rupture and conversion to open surgery [57, 69].

The current consensus is to consider intervention in persistent type II ELs when they are associated with significant sac expansion. This is commonly considered to be > 5 – 10 mm [1, 9–11, 55, 56] over 12 months [11, 55]. Type II EL with stable sac size should be managed conservatively with regular imaging follow-up as proposed in Fig. 1.

### Management

In suspected cases of type II EL associated with sac expansion, it is important to consider whether this could represent an occult type I or III masquerading as a type II. If CTA is inconclusive, catheter angiography + / − cone-beam CT may be useful to clarify the source of the EL and plan endovascular treatment, which may be performed at the same time [11].

The mainstay of treatment of type II EL is embolisation with the aim of occluding the arteries supplying the EL as well as the EL cavity itself [11, 55]. Complex type II EL with multiple supplying arteries may behave in a similar manner to a high-flow vascular malformation with a central nidus and multiple inflow and outflow vessels [11]. The choice of intervention approach and technique are dependent on the anatomy of the EL and operator experience. In some challenging cases, more than one technique may be used to achieve successful embolisation. CTA with multiplanar reformats is useful in case planning and pre-empting potential technical challenges.

#### *Transarterial *[55, 66, 72–81]

Transarterial embolisation is the most common technique that involves catheterization of the dominant feeding vessel via collateral channels. This approach is most successful in ELs involving the IMA where there is usually a long and tortuous course but one that is relatively large in calibre. Conversely, technical success may be limited in lumbar ELs where the feeding artery can be remote from the iliolumbar artery via small and tortuous branches.

**Fig. 4:**
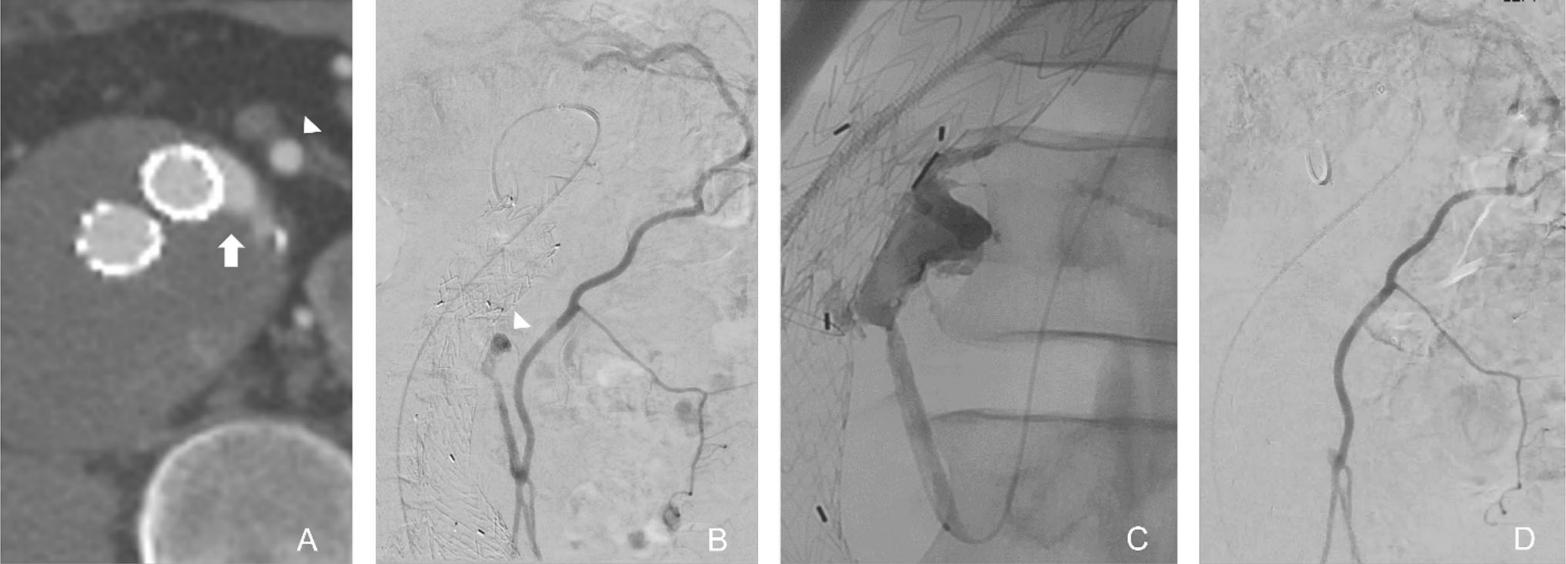
Transarterial embolisation of type II EL. (**A**) Axial CT image shows type II EL in the anterior sac (arrow) close to the IMA (arrowhead). (**B**) Angiogram from the middle colic branch of the SMA opacifies an hypertrophied arc of Riolan, IMA and EL cavity (arrowhead). (**C**) Embolisation with liquid embolic agent via a microcatheter. (**D**) Completion angiogram shows no further EL opacification

Type II EL involving the IMA may be accessed via the SMA. The usual route is as follows: SMA—middle colic artery—arc of Riolan or marginal artery of Drummond (which is usually hypertrophied)—left colic artery—IMA—aneurysm sac—EL cavity (Fig. 4). The SMA and middle colic artery are selected in turn with a 4- or 5-Fr catheter supported by a long vascular sheath. A microcatheter is then coaxially advanced along the long and tortuous route to the IMA and into the EL cavity. An angiogram is performed to outline the EL cavity and any outflow arteries, and to assess the overall flow. Embolisation is commonly performed with liquid embolic agents (e.g. NBCA, EVOH) especially when there is a large EL cavity to fill. In high-flow ELs that involve multiple arteries, metallic coils may be used to prevent inadvertent non-target distal embolisation of the liquid embolic agent. This can be achieved by embolising one or more outflow arteries or loosely packing the EL cavity with coils, which reduces the overall blood flow and therefore the degree of distal penetration of the liquid agent.

Type II EL involving lumbar arteries may be accessed via the iliolumbar artery from an ipsilateral common femoral access. Once the optimal course has been outlined on angiography, a microcatheter is advanced coaxially into the feeding lumbar artery and into the EL nidus. Angiography from the EL nidus may opacify other lumbar arteries that are involved which should also be embolised. Similarly, if there are multiple and fast-flowing arteries with a large nidus, it may be prudent to coil embolise these branches first before filling the nidus with a liquid embolic agent. If it is not possible to reach the nidus from this approach, a more proximal embolisation may be attempted either with a lower viscosity liquid agent or with coils [55]. However, this may result in recurrence of the EL from collateral vessels at other lumbar levels.

#### *Direct Sac Puncture *[82–84]

The EL nidus may be targeted directly by percutaneous puncture of the aneurysm sac. This may be performed via a transabdominal approach with the patient supine or a translumbar approach with the patient positioned prone. CTA and on-table Doppler US are used to plan a safe percutaneous route to the EL cavity avoiding bowel or large vessels and a trocar needle (18–20G) is advanced through the aneurysm sac into the EL cavity under sonographic guidance. Satisfactory position within the EL cavity is confirmed when arterial flow blood is seen from the hub of the needle and angiography is then performed to opacify the EL cavity and feeding arteries. Embolisation with a liquid embolic may be performed directly through the outer cannula of the trocar device. Alternatively, the trocar cannula may be exchanged for a 4-Fr sheath over a stiff guidewire. Large outflow vessels may be embolised with coils prior to embolisation of the nidus with a liquid agent. It may be necessary to use a microcatheter and guidewire deeper into the nidus and/or feeding vessels prior to embolisation.

#### *Transiliac Paraendograft *[85–87]

This is an adjunctive technique when the transarterial technique has been unsuccessful in reaching the EL. It may be possible to manipulate a catheter and hydrophilic guidewire into the potential space between the iliac limb endograft and arterial wall, navigating through the sac thrombus into the EL cavity. Once the position of the catheter tip within the EL cavity is confirmed on angiography, the EL is embolised with a liquid embolic agent ± coils as appropriate.

#### *Transcaval *[88–91]

This adjunctive technique involves accessing the aneurysm sac via percutaneous puncture of the inferior vena cava (IVC). With the patient positioned prone, an angled sheathed needle from a transjugular liver access set (e.g. Colapinto, Angiodynamics, USA; Rosch-Uchida, Cook, USA) is used to pierce the wall of the IVC into the adjacent aneurysm sac to reach the EL cavity. A catheter is then advanced through the sheath for subsequent embolisation. This approach may be beneficial in a small number of patients where the EL cavity is located on the right side of the aneurysm sac that is not amenable to access by other techniques.

#### Surgical

Surgical treatment options include laparoscopic clipping of aortic side branches, open surgical ligation of bleeding vessels and sac plication. Surgery is usually reserved for cases where endovascular techniques have been unsuccessful.

## Type III Endoleak

### Definition

Type III ELs arise from a structural defect of the stent graft, either secondary to a modular disconnection of its components (IIIA), a fabric tear (IIIB) or a junctional separation of fenestrated or branched stent grafts (IIIC). It may occur due to inadequate overlap of stent graft components, device migration or material fatigue.

Type III EL is relatively uncommon with a reported incidence of 0.7–4.5% [2, 4, 36, 38, 92]. First- and second-generation stent grafts are associated with a significantly higher incidence of type III EL when compared with more recent third generation devices, 12.7% and 1.2%, respectively [92]. Most fabric failures have been found to be associated with specific graft materials and designs, which have subsequently been modified or withdrawn from the market. A recent example is the earlier generation of the AFX device (Endologix, USA) that was withdrawn in 2016 after unacceptable rates of type III EL were reported and the US Food and Drug Administration intervened [93, 94].

The reported incidence of type III EL in fenestrated or branched EVAR is more variable. A higher degree of device modularity and procedural complexity does not appear to increase the incidence of type III EL as demonstrated in a large multicentre retrospective cohort study of over 4,000 cases. Type III EL remained relatively uncommon at 4% and the majority were primary ELs identified around the time of the index procedure [95]. This contrasts with the findings of single-centre series of complex EVAR where type III EL was seen in as many as 12% of patients, the majority of which were secondary EL, and type III EL was identified as the most frequent indication for re-intervention. Fenestrated EVAR (F-EVAR) with large diameter devices (34-36 mm) appear to have an increased risk of type III EL and the need for re-intervention [96].

### Indication for Treatment

Type III EL leads to increased pressure within the aneurysm sac and are associated with a risk of aneurysm rupture. They therefore warrant prompt treatment once detected [1, 9–11, 97, 98].

### Management

**Fig. 5:**
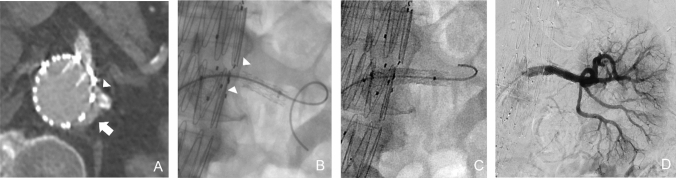
Type III EL in a F-EVAR. (**A**) Axial CT image shows junctional separation between the main body and fenestrated left renal stent (arrowhead) resulting in type III EL (arrow). (**B**) Fluoroscopic image confirms a visible gap between the two stent components (arrow heads). (**C**) Additional renal stent graft deployed to bridge the gap. (**D**) Angiogram shows no residual EL

Treatment of a type III EL may include endovascular, hybrid and open surgical techniques. Intraoperative type III ELs should be treated at the time of diagnosis, which can often be achieved by repeat balloon dilatation of areas of component overlap or by placing an additional stent graft across the separated components to bridge the gap. Similarly, the mainstay of treating secondary type IIIA or IIIC EL is by placing a bridging stent graft or aortic cuff to close the gap between the separated components [1, 92–101] (Fig. 5).

Type IIIB EL from a fabric tear may be treated by relining the main body or iliac limb. However, if the tear lies close to the flow divider, treatment becomes more complex. It may be possible to place a new bifurcated device within the existing device if there is sufficient length between the proximal landing zone and the flow divider of the existing main body to enable correct deployment of the contralateral limb of the new device. If this is not feasible, a custom-made device with an inverted iliac limb may be considered if one is available. A hybrid solution of placing an AUI device with contralateral common iliac occlusion and a surgical femoral-femoral bypass is often used in cases where there is no suitable endovascular option [101]. Finally, conversion to open surgery may be considered if these techniques fail to treat the EL [1, 9, 92, 98].

## Type IV Endoleak

Type IV EL represents leakage of blood through the stent graft due to fabric porosity in the early postoperative period. They were described mainly in first-generation stent grafts at the time of completion angiography when patients are fully anticoagulated. They are rare in newer generation devices, are self-limiting and do not require re-intervention [1, 9, 11, 12].

## Type V Endoleak

### Definition

Type V EL is also known as endotension and represents aneurysm sac expansion in the absence of an identifiable EL. This may be due to such slow blood flow that it is below the sensitivity limits for detection on current imaging methods [102–105]. For example, several authors have suggested that the vasa vasorum of the aneurysmal aortic wall may be a source of occult type II EL [106–108]. Other alternative hypotheses include hyperfibrinolysis and local coagulation activation leading to sac hygromas [109–111], ultrafiltration across the fabric of the endograft [112, 113], and pressure transmission through thrombus or the stent graft [102, 103]. It is an uncommon phenomenon reported in 2–3% [114] of EVARs and as the underlying mechanism remains contentious, each case is treated on an individual basis. Observation may be appropriate in some cases but the criteria for conservative management are unclear [2].

### Indication for Treatment

Type V EL is a diagnosis of exclusion when all other causes of sac expansion have excluded. Potential cases require additional imaging to exclude an occult EL. MRI has been shown to be more sensitive than CTA for the detection of type II EL and some authors recommend its use in patients with a suspected type V EL [31]. Catheter angiography with cone-beam CT has also been suggested as a useful imaging adjunct [11].

### Management

Treatment of type V EL is not yet defined and remains a challenge. Reported interventions include percutaneous sac aspiration, open surgical exploration and sac plication/resection, which have been unsuccessful in preventing sac enlargement [109, 110, 115]. Relining of the entire stent graft has also been described [111]. If device relining fails, then open surgical conversion may be the only viable option.

## Outcomes

### Primary Type IA

Repeated balloon moulding, giant bare metallic stents and short aortic cuff are successful in 90–100% [116–118].

EndoAnchors may be useful in challenging aortic necks but outcomes to date have not demonstrated significant benefit in reducing type IA EL when compared with non-stapled cases using latest generation of stent grafts [52, 53].

### Secondary Type IA

Early outcomes of various treatment strategies are outlined in Table 4. The choice of treatment should be based on the patient's condition, the characteristics of endoleak and the anatomy of the aorta. As suggested in a recent meta-analysis single or double chimney grafts can be an alternative to simple or fenestrated cuffs [119]. EndoAnchors have no benefit over conservative management in secondary type IA EL [119].

**Table 4:** Summary of outcomes of treatment of Type I–III EL

Endoleak type	Treatment	Success (%)	Comments
Primary IA	Balloon moulding Bare metallic stent Aortic cuff	90–100 [116–118]	
	EndoAnchor	96.5 [52]	Success = Freedom from EL at 15.4 months
Secondary IA	Chimney graft	94 [119]	Clinical success = complete resolution of EL within 30 days or significant reduction to merit surveillance without re-intervention
	Fenestrated cuff	91 [119]	
	Aortic cuff	89 [119]	
	Conservative	75 [119]	
	EndoAnchor	57 [119]	
	Embolisation	92 [119]	
IB	Graft extension	90–100 [47, 120]	
II	Transarterial	73–100 [11]	Clinical success = stable sac size
	Direct sac puncture	85–100 [11]	
	Transcaval	66–86 [11]	
III	Stent graft extension AUI Relining stent graft	75 [92]	Success = freedom from EL at 10.6 years

Transcatheter embolisation is successful in the short-term but endoleak recurrence is highly variable, between 0 and 58% at 2 years [119–129]. Therefore, embolisation should be considered in a select cohort of patients where traditional endovascular and surgical options are unsuitable or have failed.

### Type IB

Distal stent graft extension is successful in 90–100% of cases with less than 7% requiring endovascular re-intervention [47, 130]. Sacrificing the IIA can result in buttock claudication in up to 6.7% of cases, although symptoms are often transient or improve with time [130].

### Type II

There is wide variation in the reported technical and clinical success rates of type II EL embolisation [55, 72, 131, 132] from mostly retrospective single-centre series with relatively small numbers [73–76, 82–91, 108, 133–135]. Most studies report promising technical success rates of 80–100% but there is variation in how clinical outcome is defined and reported.

One of the largest series with 5-year outcome data showed transarterial embolisation with glue or coils can maintain stable sac size in 82% at 1 year but this dropped to 44% by 5 years [131]. Embolisation with coils only were more likely to require a second intervention [131] and embolisation of IMA EL (72%) yield better outcomes than lumbar EL (17%) at 2 years [77], and 7 patients experienced continued sac expansion and required conversion to open repair [77].

Despite initial success, there is a significant EL recurrence rate and delayed sac expansion. Some authors have suggested that failure to occlude the EL cavity or nidus may explain late failure rates after transarterial coil embolisation of the feeding vessel only [11, 66, 76, 136, 137].

Major complications associated with type II EL embolisation are rare but complications arising from non-target embolisation have been reported. These include pulmonary embolus secondary to glue escape via the inferior vena cava, mesenteric ischaemia secondary to IMA embolisation, lumbar radiculopathy from distal embolisation of lumbar arteries, and debilitating acute lower limb claudication [74, 78, 82, 131, 137].

### Type III

A large retrospective series identified 20 patients with type III EL, all of whom underwent endovascular treatment. Four patients (20%) suffered major periprocedural complications including lower limb ischaemia, retroperitoneal haematoma and bowel ischaemia. In addition, during a mean follow-up of 10.6 years, additional intervention was necessary in five patients (25%) for recurrent type III EL and three patients required conversion to open repair [92].

## Conclusion

ELs are common complications after EVAR and may compromise the durability of aortic repair. Long-term imaging surveillance is necessary for early detection and correct classification of ELs to guide potential re-intervention. Understanding the risk factors for ELs is important for the prevention of potential ELs, which is aided by the development of new techniques and improved stent graft designs. The majority of ELs that require treatment can be managed using endovascular techniques. A clear understanding of the EL type, and therefore its likely aetiology, is essential to guide decision-making around intervention.

## Abbreviations

EL: Endoleaks
EVAR: Endovascular aneurysm repair
CT: Computed tomography
US: Ultrasound
CEUS: Contrast-enhanced ultrasound
MRA: Magnetic resonance angiography
BMI: Body mass index
CDUS: Colour duplex ultrasound
IFU: Instructions for use
NBCA: N-butyl cyanoacrylate
EVOH: Ethylene vinyl alcohol
Ch-EVAR: Chimney endovascular aneurysm repair
AUI: Aorto-uni-iliac device
IMA: Inferior mesenteric artery
SMA: Superior mesenteric artery
IVC: Inferior vena cava
F-EVAR: Fenestrated endovascular aneurysm repair
